# *Pochonia chlamydosporia* Isolate PC-170-Induced Expression of Marker Genes for Defense Pathways in Tomatoes Challenged by Different Pathogens

**DOI:** 10.3390/microorganisms9091882

**Published:** 2021-09-05

**Authors:** Xia Zhuang, Jian-Long Zhao, Miao Bai, Xing-Xing Ping, Yan-Lin Li, Yu-Hong Yang, Zhen-Chuan Mao, Guo-Shun Yang, Bing-Yan Xie

**Affiliations:** 1 Engineering Research Center for Horticultural Crop Germplasm Creation and New Variety Breeding of Ministry of Education, College of Horticulture, Hunan Agricultural University, Changsha 410128, China; zhuangxiami@yeah.net (X.Z.); baimiao@hunau.edu.cn (M.B.); liyanlin@hunau.edu.cn (Y.-L.L.); 2 The Institute of Vegetables and Flowers, Chinese Academy of Agricultural Sciences, Beijing 100081, China; zhaojianlong@caas.cn (J.-L.Z.); pxx13663441748@163.com (X.-X.P.); yangyuhong@caas.cn (Y.-H.Y.); maozhenchuan@caas.cn (Z.-C.M.)

**Keywords:** induced defenses, jasmonic acid (JA), *Pochonia chlamydosporia*, pathogen, root endophytes, salicylic acid (SA)

## Abstract

*Pochonia chlamydosporia* is a fungal parasite of nematode eggs. Studies have shown that some strains of *Pochonia chlamydosporia* can promote plant growth and induce plants’ systemic resistance to root-knot nematodes by colonizing in their roots. This study aimed to verify the effect of the PC-170 strain on tomato growth and systemic resistance. Split-root experiments were conducted to observe the systemic resistance induced by PC-170. To explore the defense pathway that was excited due to the colonization by PC-170, we tested the expression of marker genes for defense pathways, and used mutant lines to verify the role of plant defense pathways. Our results showed that PC-170 can colonize roots, and promotes growth. We found a role for jasmonic acid (JA) in modulating tomato colonization by PC-170. PC-170 can activate tomato defense responses to reduce susceptibility to infection by the root-knot nematode *Meloidogyne incognita*, and induced resistance to some pathogens in tomatoes. The marker genes of the defense pathway were significantly induced after PC-170 colonization. However, salicylic acid (SA)- and jasmonic acid (JA)-dependent defenses in roots were variable with the invasion of different pathogens. Defense pathways play different roles at different points in time. SA- and JA-dependent defense pathways were shown to cross-communicate. Different phytohormones have been involved in tomato plants’ responses against different pathogens. Our study confirmed that adaptive JA signaling is necessary to regulate PC-170 colonization and induce systemic resistance in tomatoes.

## 1. Introduction

Plants are constantly subjected to various attacks from phytopathogenic fungi, bacteria, and plant-parasitic nematodes. Plants are not entirely passive, because the plants themselves have a series of corresponding immune responses, and they benefit from endophytic fungi to modulate their interactions with pathogens. In recent decades, some thought-provoking plant innate immune models have been put forward, such as the ‘Zigzag model’ in 2006, which indicated that there was co-evolution between pathogens and plants [1]. The ‘Invasion model’ in 2015 described the symbiotic relationship between pathogens and plants [2], while the ‘Spatial Immunity Model’ in 2019 defined immune signaling in plant–microbe interactions [3]. In addition to the induction of immunity in plants by the recognition of pathogenic effectors in host cells, plant roots benefit from endophytic and mycorrhizal fungi, which can provide plant hosts with nutrition and protection from abiotic or biotic stresses [4]. For example, fungal endophytes improve seed germination, and promote root formation and plant growth by producing plant hormones such as indole-3-acetic acid (IAA) [5,6].

*Pochonia chlamydosporia* belongs to the Clavicipitaceae family [7,8], which has a natural inhibitory effect on plant-parasitic nematodes [9,10,11]. This fungus has been applied as a promising biological control agent of nematodes in many economically important crops [9,12,13,14]. It has been reported that *P. chlamydosporia* has the ability to colonize in the roots of both monocotyledonous and dicotyledonous plants, including barley [15], tomatoes [16], potatoes [17], and *Arabidopsis thaliana* [18]. Interestingly, *P. chlamydosporia* has also exhibited multiple functions in promoting plant growth [9,19]. Previous studies have shown that colonization of *P. chlamydosporia* in plant root systems promotes growth and increases yield [20,21]. Studies have confirmed the endophytic colonization of *Arabidopsis* by *P. chlamydosporia*, and its ability to induce beneficial traits [19]. Some studies have shown *P. chlamydosporia* to induce systemic resistance to *Meloidogyne incognita* in tomatoes, but other studies have indicated that this response is plant-species-dependent [22]. Recently, based on genome and secretome analysis of *P. chlamydosporia*, a number of secreted proteins have been identified that enabled a comprehensive understanding of the endogenous mechanisms and adaptive evolution of *P. chlamydosporia* [23]. Investigation of molecular mechanisms provided evidence that *P. chlamydosporia* induces plant defenses [24].

Phytohormones play essential roles during plant growth and development, and also coordinate the modulation of plant defenses [25]. Some studies have shown that endophytes accelerate the activation of plant defense responses when different pathogens attack plants. According to the various methods that activate different signaling pathways, the microorganism-activated resistance of plants is divided into two types: One is systemic acquired resistance (SAR), which is characterized by an early increase in endogenous salicylic acid (SA) and the accumulation of pathogenicity-related (PR) proteins. The other is inducible systemic resistance, which does not involve the expression of PR proteins, but is mediated through signaling pathways, in which the plant hormones jasmonic acid (JA) and ethylene (ET) play key roles [26]. Plants protect themselves from infection by biotrophic pathogens by activating SA-dependent signals; however, against necrotic pathogens that kill plant cells, and against chewing insects, JA-dependent signaling is generally effective [27]. Plant-parasitic nematodes penetrate into plant roots, migrate into the vascular system and, finally, establish feeding sites by recruiting the plant’s developmental pathway, which involves hormonal crosstalk [28,29,30].

Around the time of hypersensitive response (HR) development, the levels of salicylic acid (SA) increase, along with the induction of various defense-associated genes, including the pathogenesis-related (*PR*) genes. The non-expressor of *PR-1* (NPR1) mutants are insensitive to SA, fail to develop SAR, and exhibit enhanced disease susceptibility. For this reason, the expression of *PR* genes is frequently used as a marker of SAR development [31]. Pathogenesis-related protein-la (*PR-1a*) is a protein that is strongly induced during the onset of systemic acquired resistance (SAR) in tobacco [32]. The *PR1b1* (also named *PR-P6*) gene is also strongly activated locally in tissues undergoing the hypersensitive response. Phenylalanine ammonia lyase (*PAL*) catalyzes the deamination of phenylalanine to trans-cinnamic acid, and is the first rate-limiting enzyme in the phenylpropanoid metabolism pathway. The expression of the *PAL* gene marks the beginning of SA biosynthesis. Silencing or epigenetically suppressing *PAL* genes inhibits the salicylate production, preventing the development of systemic acquired resistance in response to infection by the tobacco mosaic virus [33]. *NPR1*, which was identified in *Arabidopsis* through genetic screens for SAR-compromised mutants, is an important transducer of the SA signal in disease resistance, and is essential for SA-induced *PR-1a* gene expression. *NPR1* was reported as being a positive regulator of SA-dependent, defense-related gene expression, but negatively regulates JA-responsive gene expression [34]. Thus, *NPR1* modulates crosstalk between salicylate- and jasmonate-dependent defense pathways. *LoxD* and *OPR3* are JA biosynthesis genes. In the biosynthesis of jasmonic acid (JA), the lipoxygenases (*LOX*) are a group of enzymes responsible for converting both linoleic and linolenic acids into 9- and 13-hydroperoxides. *OPRs* belong to a family of flavoproteins. *OPR3* of tomato is a peroxisomal enzyme. The role of *OPR3* in JA biosynthesis has been shown by genetic evidence provided by the JA-deficient mutants *opr3* and *dde1*, both affected in *OPR3*. All genes encoding enzymes of JA biosynthesis are JA-inducible [35]. In response to wounding by insects or mechanical damage, plants synthesize multiple proteinase inhibitors with a wide range of specificities (e.g., wound-inducible serine proteinase inhibitors I and II, and cysteine proteinase protein inhibitors). Proteinase inhibitor II (*PI II*) and multicystatin (*MC*) are the typical JA-regulated, wound-related genes.

Using the split-root test in tomatoes, studies suggest that both salicylic-acid- and jasmonic-acid-dependent signaling pathways have been proposed as being responsible for systemically induced resistance to *Meloidogyne* spp. [22,36,37]. Martínez-Medina [37] proposed that *Trichoderma harzianum* T-78 first induces the upregulation of salicylic-acid-related gene expression during nematode infection, followed by the induction of jasmonic-acid-related gene expression. Some studies suggest specific and time-dependent relationships linking host plants and *P. chlamydosporia* in the presence of biotic stress factors, with colonization by *P. chlamydosporia* in plant roots being functional to a systemic, but complex, activation of defense genes [22,38]. In addition, evidence shows that jasmonic acid (JA) plays a key role in regulating root colonization by *P. chlamydosporia*. Similarly, it has been reported that *P. chlamydosporia* induces resistance to fungal disease and abiotic stress [15,39], via means such as the regulation of miRNAs in tomatoes [40], or regulation of the expression of plant defense genes relating to the salicylic acid (SA) and JA pathways in barley and *Arabidopsis* [7,18].

Therefore, further research on beneficial rhizosphere microorganisms (such as endophytes) is important. There have been studies on the interaction between *P. chlamydosporia* and other plants, but there are certain differences between different strains. *P. chlamydosporia* strain 170 (PC-170) was isolated from the root-knot nematode *M. incognita* [23]. This research aims to study the influence of PC-170 on defense systems during infection by root-knot nematodes and other pathogens. Tomato SA and JA mutant lines were used to explore the importance of defense pathways in the systemically induced resistance, which will broaden our knowledge on the working mechanisms of an important beneficial endophyte.

## 2. Materials and Methods

### 2.1. Biological Material and Growth Conditions

The PC-170 strain of *P. chlamydosporia* used in this study was isolated from *Meloidogyne incognita* eggs. The strain was stored in the CGMCC (China General Microbial Culture Collection Center) under the preservation number 8860 [41]. The PC-170 strain, and a stable green fluorescent protein (GFP)-expressing transformant (PC-170gfp) that was constructed via protoplast transformation [42], were cultured on potato dextrose agar (PDA)-medium plates in a 28 °C incubator.

The tomato (*Solanum lycopersicum*) cultivars Lichun, Castlemart, and Moneymaker were used in this study. These tomato varieties are not resistant to nematodes or various other pathogens, and are classified as susceptible varieties. Additionally, the JA-impaired mutant *def1* in the Castlemart background [43] and the SA-deficient line *NahG* in the Moneymaker background [44] were used. The *DEF1* gene acts after lipoxygenase conversion of linolenic acid to hydroperoxylinolenic acid, but before the conversion of 12-oxo-phytodienoic acid (PDA) to JA. The *def1* mutant is deficient in the *DEF1* gene; thus, the octadecanoid pathway in the *def1* mutant is blocked downstream of 13(S)-hydroperoxylinolenic acid (HPLA) action, but upstream of 12-oxo-phytodienoic acid (PDA) action. *NahG* is a gene encoding salicylate hydroxylase that removes endogenous SA by converting it to catechol. The *NahG* mutant expressing the *NahG* gene, encoding salicylate hydroxylase, did not accumulate SA in response to intercellular infiltration with washing fluid. Tomato seeds were surface-sterilized with 2% sodium hypochlorite, thoroughly rinsed with sterile water, and then germinated for 10 d in soil at 25 °C. Then, tomato seedlings were transplanted into soil in different nutrition bowls in a greenhouse according to the experimental requirements. The soil used in all experiments was made of peat soil (KLASMANN876, Germany), perlite, and vermiculite mixed in a ratio of 2:1:1, and then sterilized at 121 °C for 60 min after mixing. The plants received water according to their normal growth needs.

Tomato fusarium wilt (*Fusarium oxysporum* f.s p. *lycopersici* (Sacc.) Snyder and Hansen, TFW) was grown on PDA plates with rifampicin. Tomato bacterial wilt (*Ralstonia solanacearum* (Smith) Yabuuchi et al., TBW) was preserved in sterile distilled water. Cucumber mosaic virus (CMV) and tomato mosaic virus (ToMV) were stored in the laboratory, and were inoculated regularly on the leaves of tobacco (*Nicotiana tabacum* L.).

The long-term propagation of *Meloidogyne incognita* was carried out in a greenhouse with water spinach (*Ipomoea aquatica* Forssk.) as the growth material. We used tweezers to manually pick out the nematode eggs from the roots of the water spinach, disinfected them with 0.5% sodium hypochlorite, washed them with sterile water, collected them on a 25 μm sieve on a 90 mm plate, and stored them at 25 ± 2 °C. The second stage juveniles (J2s) were collected from the filter with a pipette every day. The number of J2s collected for use was determined according to the needs of the experiment.

### 2.2. Colonization of PC-170 on the Roots of Tomatoes

#### 2.2.1. Inoculation Methods

The tomato variety used in the experiment was Lichun. Inoculation methods included fungus cake inoculation and spore fluid inoculation. Fungus cake inoculation was performed by using a punch to remove a 1 cm cylindrical piece of fungus cake of the PC-170 strain growing on a PDA plate, which had been cultured in a 28 °C incubator for 1 week, and then evenly burying four of these 1 cm fungus cake pieces in the rhizosphere of each tomato plant. The spore fluid inoculation was conducted by using sterile water to rinse the PDA plates inoculated with the PC-170 strain. The plates were rinsed several times to collect conidia that were growing on the PDA plates after culturing in an incubator at 28 °C for 1 week. Three different concentrations of spore liquid were used for inoculation: 5 × 10^2^ spores·mL^−1^, 5 × 10^6^ spores·mL^−1^, and 5 × 10^10^ spores·mL^−1^. Each nutrient bowl was filled with 50 g of soil mixed with 20 mL of prepared spore solution before tomato transplantation.

#### 2.2.2. The Detection of Colonization

To detect endophytic colonization, the roots of three plants were randomly selected at different time periods after the inoculation with PC-170, and then the roots were rinsed and disinfected with 1% sodium hypochlorite for 1 min, washed three times in sterile distilled water, and blotted onto sterile paper. Roots were then cut into 1 cm fragments (10 fragments·plate^−1^) and plated in growth-restricting medium (PDA 39 g·L^−1^, NaCl 17.5 g·L^−1^, rose bengal 75 mg·L^−1^, Geneticin (G418) 300 mg·L^−1^, and 50 mg·L^−1^ each of streptomycin sulfate and chloramphenicol). Total root colonization by *P. chlamydosporia* was evaluated using quantitative PCR (q-PCR). DNA was extracted from non-disinfected roots to assess total colonization, and the root material was collected. Roots (1 g) were freeze-dried and then ground in liquid nitrogen. Total DNA was extracted using the Plant Genomic DNA Kit (TIANGEN BIOTECH (BEIJING) CO., Ltd., Beijing, China). The DNA was quantified using a NanoDrop 2000C spectrophotometer (Thermo Scientific, Waltham, MA, USA). In qPCR experiments, tub1 primers were used to amplify a partial region of the tubulin gene of PC-170. Reactions were performed in 20 μL with 50 ng of root DNA, 500 nM primers, and 1 × SYBR Green Master Mix (TIANGEN BIOTECH (BEIJING) CO., Ltd.). All samples were diluted in 0.01% DEPC-treated water. Reactions were performed in triplicate in a Thermal Cycling StepOnePlus using the following profile: 95 °C for 15 min, 40 cycles at 95 °C for 10 s, 60 °C for 30 s, and 72 °C for 60 s. For standard curve construction, serial dilutions were prepared from 100 ng to 10 pg of genomic DNA from *P. chlamydosporia* PC-170 in 50 ng of tomato root DNA. The cycle threshold (CT) values obtained per well containing total DNA (root DNA) were correlated with CT values in the standard curve to calculate the quantity of fungal DNA vs. total DNA.

#### 2.2.3. PC-170 and *Meloidogyne incognita* Interact in Tomatoes

After growing in nutrition bowls for 3 weeks, tomato seedlings with uniform growth were planted in 785 mL pots, each containing 50 g of soil (sterilized). Nematode inoculation was performed on the 10th day after PC-170 inoculation. In all experiments, each tomato seedling was uniformly inoculated with 1000 J2s in the soil.

#### 2.2.4. Direct Interaction

Four main treatment methods were included: (1) without any treatment (CK); (2) inoculation with strain PC-170 (PC); (3) inoculation with *M. incognita* J2s (M); and (4) inoculation with PC-170 first, and then with *M. incognita* J2s (PC+M). The tomato variety used in the experiment was Lichun. All plants were placed randomly, with 30 plants per treatment. Three tomato plants were randomly selected and their roots were washed for observation. The fresh weight of shoots (FWS), dry weight of shoots (DWS), fresh weight of roots (FWR), and dry weight of roots (DWR) of the plant were measured after 45 d. The severity of nematode infection of the plants was determined at the same time. Root systems were carefully washed with tap water. Nematode performance was analyzed by counting gall numbers on roots at each timepoint, by visual inspection. Fecundity was determined by counting the number of egg masses and the number of eggs per egg mass after 45 days. The number of egg masses was analyzed by visual inspection, and the percentage of galls showing egg masses was calculated. The number of eggs per egg mass was determined by selecting 10 egg masses randomly from each root system and soaking them in 1% bleach solution for 5 min. Then, the suspension of eggs was sieved using a 20 μm sieve. Released eggs were collected in 5 mL of water, and the number of eggs was counted with the aid of an Olympus BX51 optical microscope (Olympus Corporation, Tokyo, Japan).

#### 2.2.5. Split-Root Experiments

The roots of tomato seedlings were evenly transplanted into two adjacent nutrient bowls, each of which was filled with soil mixed with 20 mL of PC-170 spore liquid. The split-root experiments consisted of five main treatments: (1) the left and right root systems were not treated (O–O); (2) the left was pre-inoculated with PC-170, but the right was not treated (PC–O); (3) the left was challenged with *M. incognita* and the right was not treated (M–O); (4) the left was pre-inoculated with PC-170 and challenged with *M. incognita* (PC+M–O); and (5) the left was pre-inoculated with PC-170 and the right was challenged with *M. incognita* (PC–M). The tomato variety used in the experiment was Lichun. All plants were placed in a completely random design, with 30 plants per treatment. Plant height and root and shoot weight were measured after 45 days. At the same time, the severity of nematode infection of plants was determined via the methods described above. Among them, treatments (3) and (5) were observed at 15 days, 25 days, and 35 days. At six points in time (0 days, 5 days, and 10 days with PC-170, and 1 day, 15 days, and 45 days with *M. incognita*), root material was collected from tomato plants that underwent treatments (3) and (5), and then stored at −80 °C.

#### 2.2.6. Mutant Tomato Lines

The split-root experiments described above were used for all mutant tomato lines. Then, we treated the *def1* mutant tomatoes with 20 mL of 0.1 mM methyl jasmonate (MeJA) solution. MeJA solution was prepared from a 100 mM MeJA (Sigma Odridge (Shanghai) Trading Co., Ltd., Shanghai, China) stock solution in 96% (v/v) ethanol. The experiment consisted of three main treatments: (1) half of the root system was challenged with *M. incognita*; (2) the left was pre-inoculated with PC-170 and the right was challenged with *M. incognita*; and (3) the left was pre-inoculated with PC-170 and treated with MeJA, while the right was challenged with *M. incognita*. All plants were placed in a completely random design, with 30 plants per treatment and three biological replicates for each treatment. At 1 h, 6 h, 12 h, 24 h, and 48 h after the first treatment with MeJA, and on the 10th day, the colonization of PC-170 in the roots was tested using the method described above. At 45 d, the severity of nematode infection of plants was determined via the methods described above.

#### 2.2.7. PC-170 and Pathogens Interact in Tomatoes

Tomato fusarium wilt was grown on PDA plates with rifampicin. After a few days, it was inoculated in potato dextrose broth (PDB) medium and placed on a 28 °C shaker at 150 revolutions per minute (rpm) to collect spores. The spores were counted using a blood cell counter, and were then diluted to a final density of 1 × 10^6^ spores·mL^−1^ for use. Using the method of root irrigation, 15 mL of the spore solution prepared above was added to each tomato root. Tomato bacterial wilt was taken out of sterile distilled water, and then grown on TTC plates (2,3,5-triphenyltetrazoliumchloride, 0.005% v/v) for 36 h. Toxic colonies were transferred to the basic medium, and then sterile distilled water was added to prepare an inoculation suspension with a bacterial concentration of about 1 × 10^6^ cfu mL^−1^ after expanding for 36–48 h. Cucumber mosaic virus (CMV) and tomato mosaic virus (ToMV) were inoculated by weighing 2 g of diseased leaves and then adding 10 mL of phosphate buffer to grind them thoroughly, and were then coated on the surface of tomato leaves that were lightly dusted with carborundum. The experiments were all carried out on Lichun tomatoes. Treatment was carried out on the 10th day after PC-170 inoculation. Each pathogen included two treatments: (1) Only pathogens were inoculated; (2) PC-170 was inoculated in advance before pathogens were inoculated. All plants were placed in a completely random design, with 30 plants per treatment. The test was repeated at least three times, and a consistent trend was obtained. The leaves of three plants were randomly collected and stored at −80 °C. Disease assessments of CMV and ToMV were conducted throughout the experiment. Specific disease severity ratings were carried out at 10 d and 20 d. Disease severity was measured according to previous research [45]. Disease severity was measured using a 0–10 rating scale: 0 = no symptoms, 2 = mild mosaic symptoms on leaves, 4 = severe mosaic symptoms on leaves, 6 = mosaic symptoms and deformation of leaves, 8 = severe mosaic symptoms and severe deformation of leaves, and 10 = severe mosaic symptoms and deformation of leaves with stunted growth. Disease assessments of TFW were carried out at 5 and 10 days. A 0–4 disease severity scale was classified as follows: 0 = No infection, 1 = slight infection, 25% of full scale—one or two leaves became yellow; 2 = moderate infection—two or three leaves became yellow, 50% of leaves wilted; 3 = extensive infection—all plant leaves became yellow, 75% of leaves wilted, and growth was inhibited; 4 = complete infection—all plant leaves became yellow, 100% of leaves wilted, and the plant died [46]. The disease development of tomato bacterial wilt—expressed as disease incidence (DI) based on a disease index (di) on a scale of 0–4, where 0 = no wilting, 1 = 1–25% wilting; 2 = 26–50% wilting, 3 = 51–75% wilting, and 4 = 76–100% wilted or dead—was recorded 10 and 21 days after challenge [47]. Three replications were maintained for each treatment for the disease incidence study. Disease incidence includes both disease percentage and disease severity, as represented below:

Disease incidence formula:

(1) $$\mathrm{DI}\;\left(\%\right)=\frac{\sum \mathrm{scale}\;\times \;\mathrm{Numbers}\;\mathrm{of}\;\mathrm{infected}\;\mathrm{plants}\;}{\mathrm{highest}\;\mathrm{scale}\;\times \;\mathrm{Total}\;\mathrm{numbers}\;\mathrm{of}\;\mathrm{plants}}\times 100$$

#### 2.2.8. Quantitative RT-PCR Analysis

To analyze the changes in the tomatoes’ SA and JA pathways as a result of the fungi–plant–nematode interaction, we examined the marker genes involved in the SA and JA pathways. In the SA pathway, the expression of the genes *PR-P6*, *PR1a*, *PAL* and *NPR1* was detected. The tomato pathogenesis-related protein (*PR-P6*) gene is a pathogenesis-related protein induced by SA, and a direct indicator of the SA response. The gene coding for pathogenesis-related protein (*PR1a*) is a common marker of SA-regulated responses [48]. Phenylalanine ammonia lyase (*PAL*) is the first enzyme in the phenylpropanoid metabolism pathway. Non-expressor of pathogen-related genes 1 (*NPR1*) was shown to be a key regulator of SA-mediated suppression of JA signaling [49]. Regarding the JA pathway, the genes *PI II*, *MC*, *LoxD*, and *OPR3* were detected. Genes encoding proteinase inhibitor II (*PI II*) and multicystatin (*MC*) are the typical JA marker genes [49,50]. *LoxD* (lipoxygenase D) is involved in the early steps of the JA pathway, while OPR3 (12-oxophytodienoate 3 reductase) is a JA biosynthesis gene [22,48]. Primers described in Supplementary Table S1 were used to amplify the targets. We took 10 g of the roots collected in the above experiment to extract total RNA, and then inverted them into first-strand cDNA. Gene expression was analyzed by qPCR.

Total DNA was extracted using the Plant Genomic DNA Kit (TIANGEN BIOTECH (BEIJING) CO., Ltd.), while total RNA was extracted using the RNAprep Pure Plant Kit (TIANGEN BIOTECH (BEIJING) CO., Ltd.), according to the manufacturer’s instructions. First-strand cDNA was synthesized from 1 ug of total RNA using the FastKing gDNA Dispelling RT SuperMix (TIANGEN BIOTECH (BEIJING) CO., Ltd.). Real-time quantitative RT-PCR (qPCR) reactions and relative quantification of specific mRNA levels were performed using the Talent qPCR PreMix (SYBR Green) (TIANGEN BIOTECH (BEIJING) CO., Ltd.) according to the manufacturer’s instructions. Reactions were performed in triplicate in a Thermal Cycling StepOnePlus using the following profile: 95 °C for 15 min, 40 cycles at 95 °C for 10 s, 60 °C for 30 s and 72 °C for 60 s. The gene-specific primers are described in Table S1. Three technical replicas were analyzed. The data were normalized using the housekeeping gene *SlEF* (X14449), and then the relative expression levels were calculated using the 2^−ΔΔCT^ method.

#### 2.2.9. Statistical Analysis

All data were subjected to analysis of variance (ANOVA) using SPSS software (IBM, Chicago, IL, USA) to determine significant differences (*p* < 0.05) between treatments. When the data met the normal distribution and the variance was homogeneous, a paired comparison using Student’s *t*-test was carried out, or means were compared using Tukey’s test (*p* < 0.05). When ANOVA assumptions were not met, multiple comparisons were conducted using the Kruskal–Wallis test, and groups separated by Dunnett’s test (*p* ≤ 0.05). All of the experiments were repeated at least three times, with similar results.

## 3. Result

### 3.1. Pochonia chlamydosporia PC-170 Colonizes Tomato Roots and Promotes Growth

**Figure 1:**
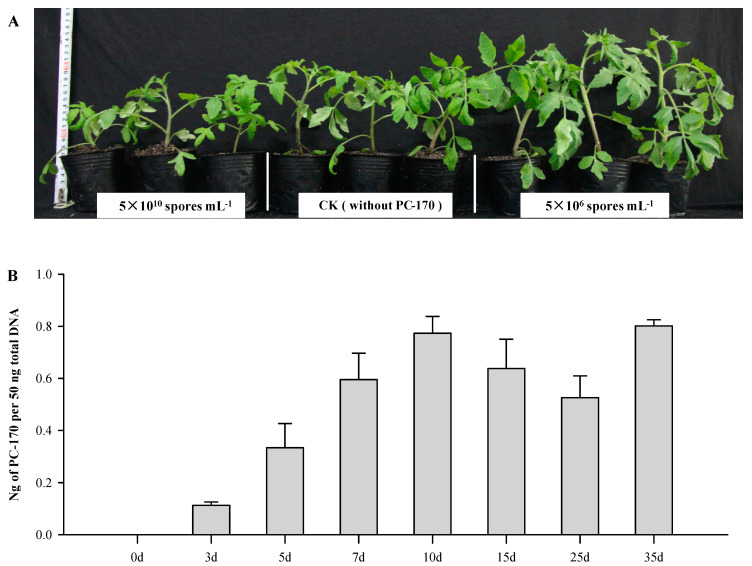
*Pochonia chlamydosporia* PC-170 colonizes tomato roots. (**A**) Tomato growth under different PC-170 spore concentrations. (**B**) The root colonization is expressed as the ratio of fungal DNA biomass per 50 ng of total root DNA. Each value is the mean ± SE of three biological samples with three technical replicates each.

It has been reported that *P. chlamydosporia* colonizes plant roots and promotes plant growth. In order to explore the effect of the *P. chlamydosporia* strain PC-170 on tomato growth, we first studied the effects of different methods used to inoculate tomato roots with PC-170. In the experiment involving the colony inoculation method, within 10 days after the inoculation of the PC-170gfp strain, no endogenous colonized hyphae were detected. In the spore liquid inoculation experiment, we observed the endogrowth of hyphae from the third day, and this phenomenon was first observed in treatment (3) (spore concentration of 5 × 10^10^ spores·mL^−1^) with the highest concentration. The growth of tomato plants was observed and compared with the untreated natural growth control group; when the concentration of spore solution was 5 × 10^6^ spores·mL^−1^, the growth of tomato plants was significantly promoted, while at high concentrations (spore concentration of 5 × 10^10^ spores·mL^−1^) tomato growth was inhibited (Figure 1A).

In order to determine the colonization process of PC-170 in tomato roots, we measured the total root colonization by *P. chlamydosporia* at different timepoints during treatment (2). The concentration of PC-170 in the roots increased over time, but eventually reached a state of equilibrium (Figure 1B). Therefore, the inoculation concentration of the spore liquid in the subsequent experiments was 5 × 10^6^ spores·mL^−1^, and other treatments were performed 10 days after inoculation (dai) with PC-170.

### 3.2. PC-170 Improves Resistance to Meloidogyne incognita

**Figure 2:**
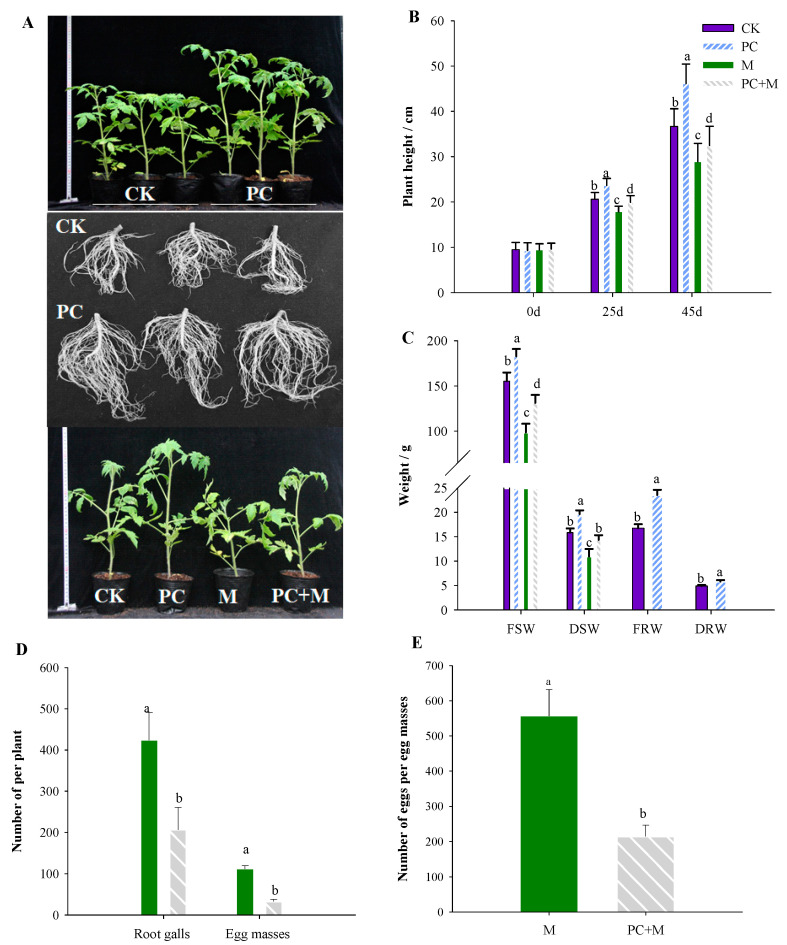
*Pochonia chlamydosporia* stimulates growth of tomato seedlings and reduces *Meloidogyne incognita* infection. CK: without any treatment; PC: inoculation with PC-170 strain; M: inoculation with *M. incognita* J2s; PC+M: inoculate first with PC-170, and then with *M. incognita* J2s. (**A**) Root growth 20 d after inoculation with PC-170. (**B**) Plant height at 0 d, 25 d, and 45 d under different treatments. (**C**) FSW (fresh shoot weight), DSW (dry shoot weight), FRW (fresh root weight), and DRW (dry root weight). (**D**) The number of root galls and the number of egg masses. (**E**) The number of eggs per egg mass. Each value is the mean ± SE (*n* = 30). Different letters indicate statistical differences between treatments (*p* < 0.05).

In the direct interaction experiment with PC-170, tomato, and root-knot nematodes, we observed that tomatoes inoculated with PC-170 grew faster and had more developed root systems than those not inoculated with PC-170. Second, all *Meloidogyne incognita*-infected tomatoes exhibited visibly restricted growth; however, this phenomenon was alleviated in plants pre-inoculated with PC-170 (Figure 2A). Additionally, the heights of tomato plants treated with PC-170 were significantly greater than those of the control group of untreated tomatoes; the difference could be observed at 25 dai and 45 dai (Figure 2B). Similarly, fresh root weight (FRW), dry root weight (DRW), fresh shoot weight (FSW), and dry shoot weight (DSW) were also significantly higher in pre-inoculated tomatoes than in the control group (Figure 2C).

We studied the control effect of the PC-170 strain on *M. incognita* -infected tomatoes. Compared with plants not inoculated with PC-170, the number of galls on the roots of plants pre-inoculated with PC-170 was reduced by 51.2%, and the average number of egg masses was reduced by 70.0%, while the number of eggs per egg mass was reduced by 58% (Figure 2D,E).

### 3.3. PC-170 Improves Resistance to Different Pathogens in Tomatoes

**Figure 3:**
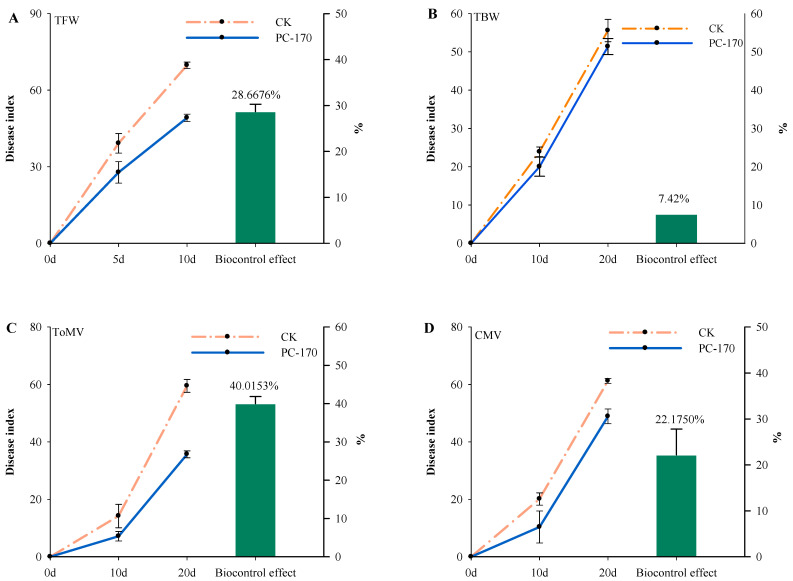
*Pochonia chlamydosporia* increases tomatoes’ resistance to different plant pathogens. (**A**) Percentage disease level at 5 d and 10 d, and biocontrol effect, after tomato fusarium wilt (*Fusarium oxysporum* f. sp. *Lycopersici* (Sacc.) Snyder and Hansen; TFW) inoculation. (**B**) Percentage disease level at 10 d and 20 d, and biocontrol effect, after tomato bacterial wilt (*Ralstonia solanacearum* (Smith) Yabuuchi et al.; TBW) inoculation. (**C**) Percentage disease level at 10 d and 20 d, and biocontrol effect, after tomato mosaic virus (ToMV) inoculation. (**D**) Percentage disease level at 10 d and 20 d, and biocontrol effect, after cucumber mosaic virus (CMV) inoculation. Three replications were maintained for each treatment for disease incidence study.

Furthermore, we studied the resistance induced by PC-170 in tomatoes to different plant pathogens. Susceptible tomato plants showed symptoms within 10 or 20 dai with pathogens. At 10 days after inoculation with TFW, 16.8% of tomato plants in the control group showed severe symptoms, while only 3.3% of PC-170-pre-inoculated plants showed severe symptoms; tomatoes’ resistance to TFW was increased by 28.7% through the action of PC-170 (Figure 3A); therefore, PC-170 played a positive role. However, PC-170 did not show a significant effect on tomatoes inoculated with tomato bacterial wilt (TBW), where there was no significant difference between the two treatments. Judging from the disease index, pre-inoculation with PC-170 did not reduce the incidence of TBW (Figure 3B). In the ToMV experiment, we observed similar results. Twenty days after viral inoculation, in the control group, 33.3% of plants exhibited severe symptoms, 56.7% of plants exhibited moderate symptoms, and 10.0% of plants showed weak symptoms. However, in the PC-170-pre-inoculated group, 10.0% of plants had severe symptoms, 66.6% of plants had moderate symptoms, and 23.3% plants showed weak symptoms; tomatoes’ resistance to ToMV was increased 40.0% by PC-170 (Figure 3C). Obvious viral symptoms were observed in 46.7% of tomato plants not inoculated with PC-170 at 10 dai with CMV, while only 13.3% of plants pre-inoculated with PC-170 showed obvious viral symptoms. After 20 d, 36.7% of the non-inoculated plants exhibited severe symptoms, with moderate symptoms in 63.3%. In the PC-170-pre-inoculated group, 13.3% of plants exhibited severe symptoms, 76.7% of plants displayed moderate symptoms, and 10% of the plants showed weak symptoms. Inoculation with PC-170 resulted in a 22.2% increase in tomatoes’ resistance to CMV (Figure 3D).

### 3.4. PC-170 Induces Systemic Resistance in Tomatoes

**Figure 4:**
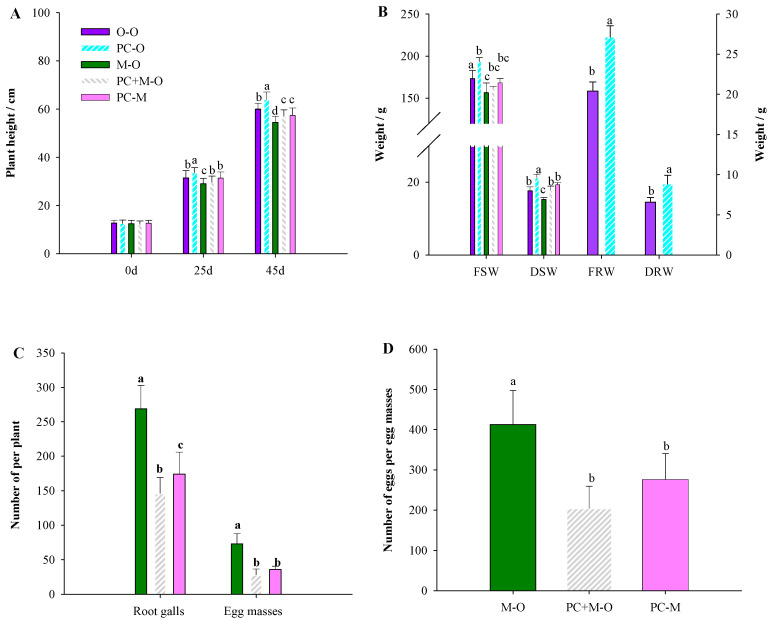
PC-170 improves tomatoes’ resistance to *Meloidogyne incognita.* O–O: The left and right root systems were not treated. PC–O: Just the left root system was pre-inoculated with PC-170. M–O: Just the left root system was challenged with *M. incognita*. PC+M–O: The left root system was pre-inoculated with PC-170 and challenged with *M. incognita*. PC–M: The left root system was pre-inoculated with PC-170 while the right was challenged with *M. incognita*. (**A**) Plant height at 0 d, 25 d, and 45 d under different treatments in split-root experiments. (**B**) Split-root experiments. FSW: fresh shoot weight; DSW: dry shoot weight; FRW: fresh root weight; DRW: dry root weight. (**C**) In the split-root experiment, the number of root galls and the number of egg masses. (**D**) In the split-root experiment, the number of eggs per egg mass. Each value is the mean ± SE (*n* = 30). Different letters indicate statistical differences between treatments (*p* < 0.05).

To study the systemic resistance induced by *P. chlamydosporia* isolate PC-170 in tomatoes, split-root experiments were conducted. In the split-root experiments, the growth of the plant was observed as in the previous experiments, and the results showed that PC-170 promoted tomato growth, while root development was not affected by the split-root system (Figure 4A,B). The results showed that tomatoes pre-inoculated with PC-170 exhibited reduced numbers of root galls, egg masses, and eggs per egg mass; taking the treatment group M–O as a control, the treatment group PC+M–O showed decreases in these metrics of 45.7%, 61% and 50%, respectively, while for the treatment group PC–M the decrease was 35.3%, 50%, and 48%, respectively (Figure 4C,D).

**Figure 5:**
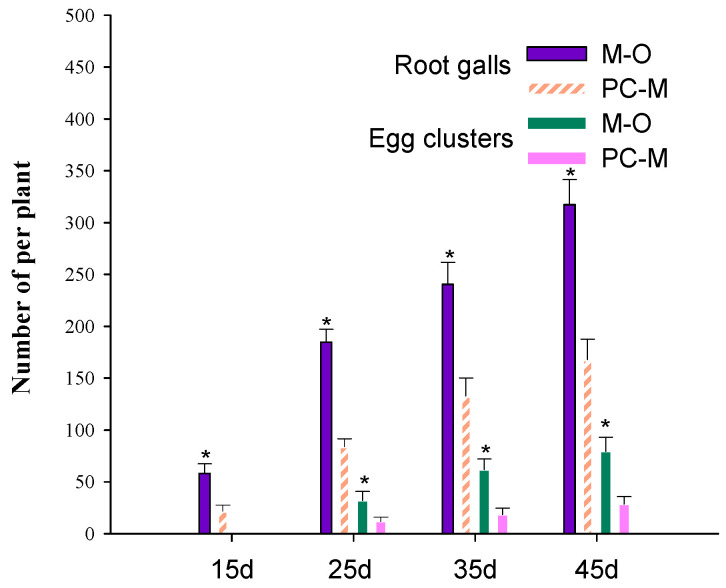
Comparison between the two treatments. M–O: Just the left root system was challenged with *Meloidogyne incognita*. PC–M: The left root system was pre-inoculated with PC-170, while the right was challenged with *M. incognita*. Each value is the mean ± SE (*n* = 10). * indicates statistical differences between treatments (*p* < 0.05).

The effect of PC-170 on the reproduction of *M. incognita* in tomato roots was investigated at 15, 25, 35, and 45 dai. From 15 to 45 dai, a significant reduction in the number of root galls was observed in the roots of plants treated with PC-170; no egg masses were observed at 15 dai, and there was a significant reduction in the number of egg masses in the plants pre-inoculated with PC-170 at 25 and 45 dai (Figure 5).

### 3.5. Expression of Defense Pathway Marker Genes in Different Interactions

**Figure 6:**
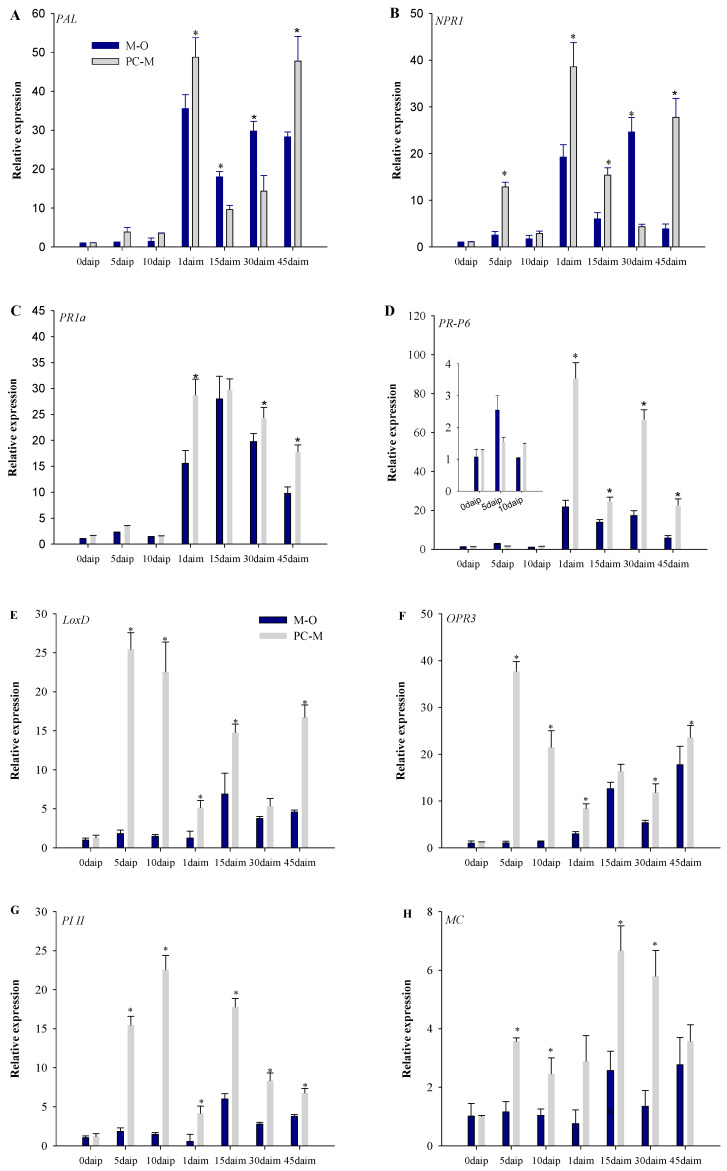
The expression changes of the salicylic acid (SA) marker genes and the jasmonic acid (JA) marker genes. M–O: Just the left root system was challenged with *Meloidogyne incognita*. PC–M: The left root system was pre-inoculated with PC-170, while the right was challenged with *M. incognita*. daip: days after inoculation with PC-170; daim: days after inoculation with *Meloidogyne incognita*. (**A**) The expression of the SA biosynthesis gene *PAL*. (**B**) The expression of the SA-dependent, defense-related gene *NPR1*. (**C**) The expression of the SA-responsive marker gene *PR1a*. (**D**) The expression of the SA-responsive marker gene *PR-P6*. (**E**) The expression of the JA biosynthesis gene *LoxD*. (**F**) The expression of the JA biosynthesis gene gene *OPR3*. (**G**) The expression of the JA-regulated, wound-related gene *PI II*. (**H**) The expression of the JA-regulated, wound-related gene *MC*. Each value is the mean ± SE of three biological samples with three technical replicates each. * indicates statistical differences between treatments (*p* < 0.05).

Before inoculation with *Meloidogyne incognita*, we observed that in the SA pathway, compared with the plants not inoculated with PC-170, the expression of the *PAL* gene in the plants inoculated with PC-170 did not increase significantly (Figure 6A). The expression of the *NPR1* gene was increased significantly at 5 days after inoculation with PC-170 (daip) (Figure 6B). Similarly, the expression of the *PR1a* and *PR-P6* genes did not change significantly (Figure 6C,D). However, in the JA pathway, the expression levels of the *LoxD* and *OPR3* genes increased significantly at at 5 daip and 10 daip (Figure 6E,F). Compared with the levels of genes at 5 daip, the amount of expression was lower again at 10 daip. High expression levels of the induced genes *PI II* and *MC* were also observed at 5 daip and 10 daip. It can be seen that the colonization of PC-170 activated the JA pathway (Figure 6G,H).

After inoculation with *Meloidogyne incognita*, we observed the expression of each gene in the plants of the M–O treatment group, and found that the expression level of the *PAL* gene increased significantly at 1 days after inoculation with *Meloidogyne incognita* (daim). Compared with the expression level at 1 daim, the expression level decreased at 15 daim, then increased again at 30 daim, and decreased once more at 45 daim (Figure 6A). The expression trends of the *NPR1*, *PR1a*, and *PR-P6* genes were also consistent (Figure 6B,C,D). In the JA pathway, the expression of the *LoxD* and *OPR3* genes decreased at 1 daim and 30 daim, and increased at 15 daim and 45 daim (Figure 6E,F). The expression of the inducible genes *PI II* and *MC* increased significantly at 15 daim (Figure 6G,H). Therefore, we can conclude that the SA pathway was the first to be activated in nematode infection. The JA pathway was also activated during the development of the nematodes in the roots (15 daim), and then the SA JA pathways appeared alternately during the time period when the nematode infestation returned (30–45 daim).

Taking the M–O treatment group as a control, we can see that after nematode infection, the expression of the SA pathway marker genes increased more significantly in the PC–M treatment. At each stage, the expression was significantly higher than that in the M–O treatment group. In the JA pathway, nematode infection caused the expression of marker genes to drop rapidly, but then it increased again at 15 daim. Consistent with the SA approach, the changes in gene expression in the PC–M treatment group were significantly greater than in the M–O treatment group. Moreover, the gene expression of the JA pathway was not completely suppressed.

**Figure 7:**
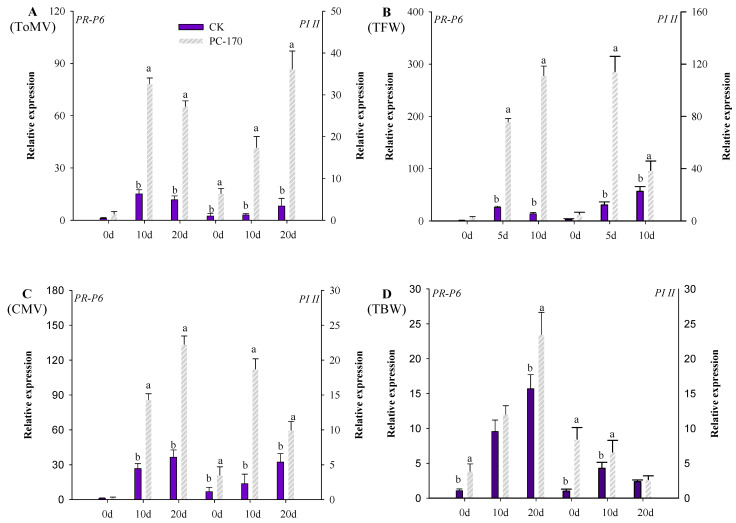
The expression of genes when PC-170 and pathogens interact in tomatoes. (**A**) The changes in the expression of the *PR-P6* and *PI II* genes during pathogenesis after ToMV inoculation. (**B**) The changes in the expression of the *PR-P6* and *PI II* genes during pathogenesis after TFW inoculation. (**C**) The changes in the expression of the *PR-P6* and *PI II* genes during pathogenesis after CMV inoculation. (**D**) The changes in the expression of the *PR-P6* and *PI II* genes during pathogenesis after TBW inoculation. Each value is the mean ± SE of three biological samples with three technical replicates each. Different letters indicate statistical differences between treatments (*p* < 0.05).

When PC-170 and plant pathogens interacted in tomatoes, we detected the marker genes involved in the SA and JA pathways. When PC-170 and ToMV interacted in tomatoes, it could be observed that the *PR-P6* and *PI II* genes were activated. In the pre-inoculated PC-170 tomato leaves, it was found that the *PL-II* gene had been activated and expressed. After inoculation with ToMV, the expression of the gene continued to be upregulated; however, the expression of the *PR-P6* gene was significantly upregulated at 10 dai, and decreased at 20 dai (Figure 7A). When PC-170 and TFW interacted in tomatoes, the expression of the *PR-P6* and *PI II* genes was activated after inoculation with TFW. In tomatoes pre-inoculated with PC-170, the fold and duration of gene upregulation were enhanced (Figure 7B). In the process of PC-170 and CMV interacting in tomatoes, the *PR-P6* and *PI II* genes were activated. However, the expression of different genes was upregulated at different time periods. It can be seen that the upregulation fold of genes in tomatoes pre-inoculated with PC-170 was significantly higher than that in non-inoculated tomatoes (Figure 7C). When tomatoes were inoculated with TBW, expression of the *PR-P6* and *PI II* genes was activated. The expression of the *PR-P6* gene was significantly upregulated, while the expression of the *PI II* gene was first upregulated and then downregulated. In the tomatoes pre-inoculated with PC-170, the gene expression pattern was consistent with that of the control group; however, the gene expression advantage induced by PC-170 was not reflected (Figure 7D).

### 3.6. PC-170 and Meloidogyne incognita Interact in Mutant Tomato Lines

To further analyze the roles of SA and JA signaling in the promotion of growth and protective effects induced by PC-170, nematode infection was assessed in the *NahG* mutant and in the *def1* mutant, as well as their corresponding background wild-type tomato lines. First, we found that not only in the Lichun tomatoes, but also in the Castlemart and Moneymaker tomato varieties, PC-170 showed a growth-promoting effect. Remarkably, the growth-promoting effect was lost in the def1 mutant, and the tomato plants’ heights were significantly lower than those of the control group that were not inoculated with PC-170. Similarly, the fresh root weight (FRW), dry root weight (DRW), fresh shoot weight (FSW), and dry shoot weight (DSW) were also significantly lower compared to those in the control group (Table S2).

**Figure 8:**
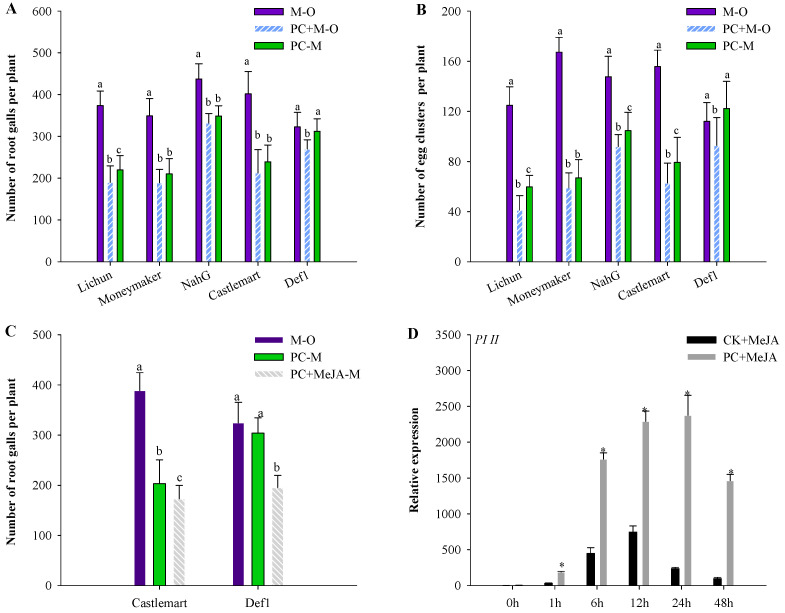
*Pochonia chlamydosporia*-induced resistance against *Meloidogyne incognita* in different tomato lines (**A**) The number of root galls was monitored at 45 d after inoculation with *M. incognita*. (**B**) The number of egg clusters in the roots was monitored at 45 d after inoculation with *M. incognita*. Data are the mean ± SE (*n* = 30). (**C**) The number of galls was monitored at 45 d after exogenous application of methyl jasmonate (MeJA). Data are the mean ± SE (*n* = 30). Each value is the mean ± SE of three biological samples. Different letters indicate statistical differences between treatments (*p* < 0.05). Each value is the mean ± SE (*n* = 30). (**D**) *PI II* was analyzed at six time points after exogenous application of methyl jasmonate (MeJA). Each value is the mean ± SE of three biological samples with three technical replicates each. * indicate a significant difference from non-PC-170-inoculated plants according to Dunnett’s test (*p* < 0.05; *n* = 5).

In the background wild-type tomato lines (Castlemart and Moneymaker), the root galls of plants were reduced by approximately 50% in the PC+M−O treatment group, and by approximately 40.0% in the PC−M treatment group, compared with the control M−O treatment group. However, in SA-deficient *NahG* tomato plants, the root galls of the PC+M−O treatment group were reduced by 24%, while in the PC−M treatment group they were reduced by 20%, compared with the control M−O treatment group. Compared with the Moneymaker background line, the effect of PC-170 was noticeably limited in the *NahG* mutant. In the JA-deficient *def1* mutant, compared with the control M−O treatment group, the root galls of the PC + M−O treatment group were reduced by 16%, while in the PC−M treatment group they were reduced by 3%. Compared with the Castlemart background line, the effect of PC-170 was significantly reduced in the *def1* mutant. Similarly, statistical analysis of the number of egg clusters per plant found that this systemic protection was reduced in SA-deficient *NahG* tomato plants. In contrast to *NahG* plants, this systemic protection was almost entirely lost in the JA-deficient *def1* mutant (Figure 8A,B).

Next, the ability of PC-170 to activate JA signaling after exogenous hormone application was investigated. Proteinase inhibitor II (*PI II*) gene expression was induced by exogenously applying methyl jasmonate (MeJA) to the roots. The induction of the *PI II* gene in plants pre-inoculated with PC-170 was stronger and more durable (Figure 8D). These results suggest that PC-170 primes JA-dependent defense signaling in systemic root tissue, which probably affects the inhibitory effect on JA-mediated signaling by *M. incognita*. We also treated the JA-deficient *def1* mutant with MeJA to investigate whether it could regain induced resistance (Figure 8C). The results showed that the JA-deficient *def1* mutant could regain resistance to nematode infection in PC-170-pre-inoculated plants as a result of spraying with the exogenous hormone MeJA.

**Figure 9:**
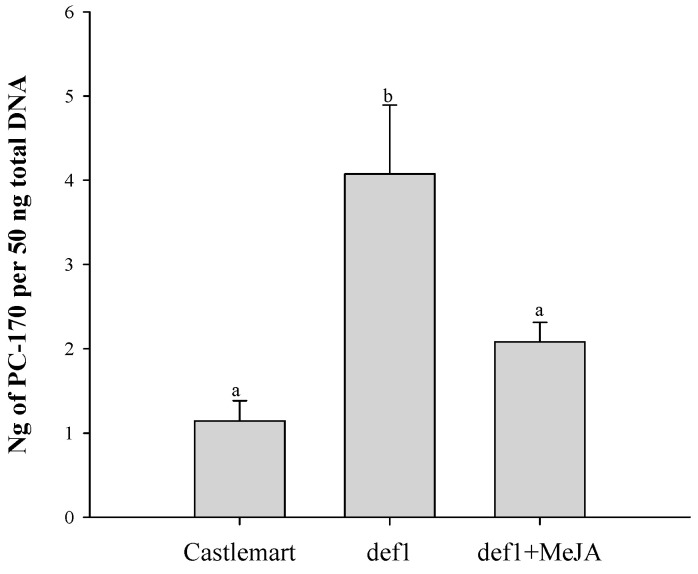
The colonization of PC-170 in the roots under the following different conditions: Castlemart: Castlemart tomatoes inoculated with PC-170; def1: *def1* mutant tomatoes inoculated with PC-170; def1+MeJA: *def1* mutant tomatoes inoculated with PC-170 and treated with the exogenous hormone MeJA. Each value is the mean ± SE (*n* = 30). Different letters indicate statistical differences between treatments (*p* < 0.05).

We compared the colonization of PC-170 in the roots under the following different conditions: (1) Castlemart tomatoes inoculated with PC-170; (2) *def1* mutant tomatoes inoculated with PC-170; (3) *def1* mutant tomatoes inoculated with PC-170 and treated with the exogenous hormone MeJA. We collected roots to detect the total root colonization after 10 days. Compared with the total colonization of PC-170 in Castlemart tomatoes, the total colonization of PC-170 in *def1* mutant tomatoes increased significantly. However, the total colonization of PC-170 was reduced when *def1* mutant tomatoes were treated with the exogenous hormone MeJA (Figure 9).

## 4. Discussion

Plants, unlike other forms of life, cannot move to escape biotic or abiotic stresses. However, plants have evolved alongside microbes. The plant microbiome is a basic and important partner in protecting the plant from stresses, in that it not only synthesizes enzymes or metabolites and produces important phytohormones that can negatively affect plant pathogens and ensure the tolerance of the plant to environmental stress [51], but also induces systemic resistance in plants by colonizing their roots [52]. These organisms that live in healthy plant tissues but do not cause disease are called endophytes. Most bacterial and fungal endophytes benefit plants by promoting growth or suppressing pathogens, and some can even improve the stress tolerance and immunity of plants [51]. Cooperation between host plants and endophytes is one of the strategies that assist plants in adapting, surviving, and developing.

It has been reported that *Pochonia* spp. can colonize roots; acting as a root endophyte, their capacity to colonize and proliferate in roots greatly varies between isolates [22,53,54]. Studies have shown that *P. chlamydosporia* has multiple functions in promoting plants’ growth and increasing their yield or promoting flowering when colonizing their roots [18] Our results indicate that the *P. chlamydosporia* isolate PC-170 colonizes and proliferates in tomato roots. Isolate PC-170 exerts a positive effect on tomato plant growth by promoting root development and increasing tomato growth under greenhouse conditions. Previous evidence also indicated that endophyte over-colonization could limit plant growth through an excess of nutrients transferred to fungal partners [18]. An effective innate immunity system was exploited to avoid root over-colonization by beneficial endophytes that may affect development [7,55]. Immune responses were induced by their progressive reduction at later stages when plants perceive beneficial fungi [52,56]. Likewise, we found that the change in JA signaling in PC-170 pre-inoculated plants conforms to this scenario. Our results showed that PC-170 in the root system would not increase after reaching a certain amount, and the gene *PL-II* was upregulated at 5 and 10 dai with PC-170. Interestingly, when using the PC-170 spore liquid inoculation method, low-concentration spore liquid grows slowly in the soil, but too high a concentration of spore liquid inhibits the growth of tomato plants. We speculate that PC-170 is a dose-dependent beneficial endophyte. In the JA-deficient *def1* mutant (Castlemart) tomatoes, the effect of growth promotion by PC-170 was lost. Numerous reports showed that JA signaling was involved in carbohydrate partitioning and limiting colonization [18,55,57]. There is also evidence showing that plant defense responses contribute to restricting beneficial endophytic colonization by modulating JA signaling [58]. In tomatoes, JA signaling affects the colonization of mycorrhizal fungi by modifying the expression of some downstream genes involved in carbohydrate partitioning [59]. Our results suggest a role for JA in modulating the colonization of tomatoes by PC-170, and also confirm that adaptive JA signaling is necessary to regulate PC-170 colonization and its benefits. Thus, in the JA-deficient *def1* mutant (Castlemart) tomato plants, the colonization of PC-170 was not effectively controlled by the plant itself, leading to the inhibition of plant growth. Studies have shown that the colonization of *P. chlamydosporia* on barley roots can upregulate phytohormone-synthesis-related genes, such as auxin, ethylene, and jasmonic-acid-synthesis-related genes [7]. It is very possible that, in this interaction, auxin-synthesis-related genes are also upregulated so that PC-170 can exert a growth-promoting effect; this point requires further confirmation.

*P. chlamydosporia*, which is a nematode egg parasitoid fungus with worldwide distribution, has been applied to the biological control of nematodes in many economically important crops [9,12,13,14,15]. The study of *P. chlamydosporia* in lettuce, cucumbers, tomatoes, and bananas also revealed that it can reduce the infection rate of nematodes [60]. In this study, PC-170 increased tomatoes’ resistance to root-knot nematodes by reducing the number of root galls and egg masses per plant. PC-170 not only affects infection by nematodes, but also affects the reproduction of nematodes within the roots. We also found that PC-170 grants tomato plants advantages, including helping them to mitigate growth inhibition by nematodes. Thus, PC-170 can be used as a biological control agent to control nematodes.

Many studies have proven that endophytic fungi are able to protect plants against a range of soil-borne pathogens. In studies of root endophytism, researchers found that a large proportion of fungal root endophytes are antagonistic to important root pathogens such as wilt and take-all fungi [61]. *P. chlamydosporia* plays an active role in suppressing plant pathogens. For example, colonization by *P. chlamydosporia* reduced the occurrence of other fungi in the roots [15]. Some study provides evidence that three nematophagous fungi can also reduce root colonization and root damage by fungal pathogens; *P. chlamydosporia* was included. In Petri dish experiments, *P. chlamydosporia* reduced colonization of barley roots by *Gaeumannomyces graminis* var. *tritici* (Ggt), and also reduced necrotic symptoms [20]. In our research, we found that PC-170 played a positive role in increasing tomatoes’ resistance to TFW. However, PC-170 did not show a significant effect on tomatoes inoculated with tomato bacterial wilt (TBW); judging from the disease index, pre-inoculation with PC-170 did not reduce the incidence of TBW. Therefore, PC-170 cannot play a positive role in the fight against all pathogens. In addition to soil-borne pathogens, we also found that PC-170 affects tomatoes’ resistance to viral diseases on leaves. CMV disease severity ratings were significantly lower for PC-170-treated tomato plants than for control plants at 10 and 20 dai. In the ToMV experiment, we also observed similar results. Moreover, we found that a delayed onset time can be observed when PC-170 interacts with pathogens in tomatoes. The results show that the colonization of tomato roots by PC-170 can induce systemic protection against foliar pathogens.

*Trichoderma atroviride* (Ta) significantly reduces the number of galls and adult nematodes inside tomato roots. Ta induces systemic resistance against *Meloidogyne javanica* (Mj), as demonstrated by an in vivo split-root experiment, without the need for the organisms to be in direct contact [36]. In order to prove the defensive mechanism of *P. chlamydosporia* in the fungus–plant interaction, study of split-root experiments demonstrates that the differential *P. chlamydosporia* isolates induce systemic resistance against *M. incognita* in tomatoes, but not in cucumber; thus, whether *P. chlamydosporia* can induce systemic resistance against root-knot nematodes depends on the plant species [22]. We also observed that plant-mediated resistance induced by *P. chlamydosporia* impacted the nematode parasitism in the split-root experiments, in a manner distinct from the possible direct nematicidal effects of the PC-170 fungus [62]. *P. chlamydosporia* induced plant defense mechanisms in fungus–plant interaction studies.

*Trichoderma* species can protect plants from pathogens or insect attack by inducing defense responses [63]. There are multiple transduction pathways that regulate *Trichoderma*-induced resistance, and the response ability of plants inoculated with *Trichoderma* species against multiple pathogens was improved by the ability to alternately activate different resistance-regulatory mechanisms [64,65]. *Trichoderma* priming of SA- and JA-dependent defenses in roots was plastic and adaptive to the parasitism stage. *Trichoderma* primed SA-regulated defenses to limit nematode root invasion, and enhanced JA-regulated defenses, which compromised galling and fecundity [37]. In 2015, researchers found an upregulation of genes implicated in the biosynthesis of plant hormones—such as auxin, ethylene and jasmonic acid—by analyzing the barley root transcriptomic response to *P. Chlamydosporia* colonization of roots [7]. Study of *P. chlamydosporia*-induced plant resistance by the split-root method revealed that the expression of the salicylic acid pathway (*PR-1* gene) and the jasmonate signaling pathway (*Lox D* gene) in tomato roots was induced both after being inoculated with the fungal isolate and after nematode inoculation [22]. In this study, we found that the relevant marker genes of the jasmonate signaling pathway were the first to be activated. As mentioned above, the JA pathway was involved in the regulation of the colonization of *P. chlamydosporia*; after inoculation with nematodes, the relevant marker genes of the SA pathway were activated. We found that the SA and JA pathways exhibited a certain antagonism, but at a certain point in time showed a synergistic effect in the process of resistance to nematodes, by analyzing the expression of related genes of the two pathways at the stage of nematode growth and development, which is a plastic phenomenon similar to that revealed by research on the anti-nematode *Trichoderma*. During the period from the first infestation of nematodes to the occurrence of the second infection, both the SA pathway and the JA pathway are induced and activated. However, the colonization of PC-170 in the roots makes this induction more rapid and intense, so it can increase the resistance of tomatoes to nematodes. Kammerhofer [30] found that jasmonic acid triggers early defense responses against *Heterodera schachtii*, but during later syncytium and female development, salicylic acid seems to be a negative regulator. In the study of PC-170 inducing tomato resistance to pathogens, the activation of defense signaling pathways also occurred. When analyzing the expression of marker genes, it was found that different pathogens activate the SA and JA pathways in different orders, which is related to different pathogens’ different mechanisms of action on tomatoes. Among the pathogens to which PC-170 plays an active role in resistance, we found that the activation of the signaling pathway became more intense, and the fold and duration of gene upregulation were improved.

We used mutant lines to verify the PC-170-induced resistance in tomatoes. In 2017, Martinez-Medina et al. showed that the systemic protection induced by *T. harzianum* was still able to protect the roots of the JA-deficient def1 mutant against nematode invasion, but was lost in SA-deficient *NahG* plants [37]. We used mutant tomato lines to analyze the effects of pathways on PC-170-induced resistance. The difference from *T. harzianum* was that we found that PC-170-mediated plant resistance against nematode invasion was restricted in *NahG* plants. Thus, we can conclude that the induction of SA-dependent defenses by PC-170 during *M. incognita* infection putatively contributed to plants’ resistance to nematodes. However, PC-170-mediated plant resistance to nematode invasion was completely blocked in *def1* plants, while the JA-deficient *def1* mutant could regain resistance to nematode infection in PC-170-pre-inoculated plants after spraying with the exogenous hormone MeJA; this is in contrast to the results of some other studies [22,37]. The JA pathway seems to play an indispensable role in tomatoes’ resistance to nematodes. In the absence of the SA pathway, there may be other hormones or synthetic pathways that can play the same role and act as a substitute for SA. The lack of JA has an impact on plants not only in terms of inducing resistance, but also in terms of growth. It may be precisely because this effect is multifaceted that it highlights the importance of JA to plants. All in all, we can know that both the SA and JA signaling pathways play roles in the process of PC-170-induced resistance to root-knot nematodes in tomatoes. Defense pathways play different roles at different points in time. In the process of PC-170 colonization, the JA pathway plays a role in regulating the colonization. When roots are infested by nematodes, the SA pathway is the first to play a role, while the JA pathway mainly plays a role during the reproduction of the nematode.

## 5. Conclusions

In conclusion, this study confirmed that *P. chlamydosporia* colonizes and proliferates in tomato roots. Isolate PC-170 had a positive effect on tomato growth, promoted root development, and increased tomato growth under greenhouse conditions. PC-170 increased tomatoes’ resistance to root-knot nematodes and induced systemic resistance. The JA pathway of plants regulates the colonization of PC-170; then, the JA pathway cooperates with the SA pathway to induce resistance to *M*. *incognita* and other pathogens in tomatoes. One of the challenges for the future will be the establishment of assays to unravel the regulatory mechanism of endophytes, and to show how PC-170 orchestrates downstream responses during interactions with root cells.

## Data Availability

The data that supports the findings of this study are available in the supplementary material of this article.

## Supplementary Materials

The following are available online at https://www.mdpi.com/article/10.3390/microorganisms9091882/s1. Table S1. Primer sequences used for real-time qPCR analysis [66,67,68,69]. Table S2. The effect of the PC-170 strain of *Pochonia chlamydosporia* on the growth of different tomato species in two split-root experiments.
