# Supplementary material for: Pochonia chlamydosporia Isolate PC-170-Induced Expression of Marker Genes for Defense Pathways in Tomatoes Challenged by Different Pathogens
Source: Microorganisms. 2021 Sep 5;9(9):1882. doi: 10.3390/microorganisms9091882 (PMC8470021; doi:10.3390/microorganisms9091882)
Supplement: Supplementary file 1 [file microorganisms-09-01882-s001.zip › microorganisms-1345664-supplementary.pdf]

Pochonia chlamydosporia Isolate PC-170-Induced Expression of Marker Genes for Defense Pathways in Tomatoes Challenged by Different Pathogens

Xia Zhuang [1,2,†], Jian-Long Zhao [2,†], Miao Bai [1,†], Xing-Xing Ping [2], Yan-Lin Li [1], Yu-Hong Yang [2], Zhen-Chuan Mao [2], Guo-Shun Yang [1,*] and Bing-Yan Xie [2,*]

Supplementary Materials:

Table S1. Primer sequences used for real-time qPCR analysis.

Table S2. The effect of the PC-170 strain of *Pochonia chlamydosporia* on the growth of different tomato species in two split-root experiments.

Table S1. Primer sequences used for real-time qPCR analysis.

ID	Target Gene	Primer (5′–3′)
X14449	Elongation factor 1α (*SlEF*) [66]	GATTGGTGGTATTGGAACTGTC<br>AGCTTCGTGGTGCATCTC
544123	Pathogenesis-related protein (*PR-P6*) [67]	GTACTGCATCTTCTTGTTTCCA<br>TAGATAAGTGCTTGATGTGCC
M69247	Pathogenesis-related protein (*PR1a*) [48]	ATGTGTGTGTTGGGGTTGGT<br>ACTTTGGCACATCCAAGACG
K03291.1	Proteinase inhibitor II (*PI II*) [49]	CCTATTCAAGATGTCCCCGTTC<br>GGGCAATCCAGAAGATGG
AF083253	Multicystatin (*MC*) [37]	GAGAATTTCAAGGAAGTTCAA<br>GGCTTTATTTCACACAGAGATA
TC165415	Phenylalanine ammonia lyase (*PAL2*) [68]	TGAAGGAATGGAATGGTGCT<br>TGAAAGAAGCCACAAAAGTTCA
AY640378	Non-expressor of pathogen-related genes 1 (*NPR1*) [49]	GGGAAAGATAGCAGCACG<br>GTCCACACAAACACACACATC
U37840	Lipoxygenase D (*LoxD*) [69]	GACTGGTCCAAGTTCACGATCC<br>ATGTGCTGCCAATATAAATGGTTCC
AJ278332	12-Oxophytodienoate 3 reductase (*OPR3*) [69]	TTGGCTTAGCAGTTGTTGAAAG<br>TACGTATCGTGGCTGTGTTACA
28844336	Tubulin gene tub1	TTTGCAGTATCTCAGTGTTC<br>ATGCAAGAAAGCCTTGCGAC

Table S2. The effect of the PC-170 strain of *Pochonia chlamydosporia* on the growth of different tomato species in two split-root experiments.

		Plant Height (cm)	FSW (g)	DSW (g)	FRW (g)	DRW (g)
**Lichun**	O–O	65.73 ± 9.69a	193.84 ± 7.13a	20.32 ± 1.20a	21.61 ± 1.20a	8.40 ± 0.76a
	PC–O	76.23 ± 11.5b	234.33 ± 8.43b	23.66 ± 1.10b	28.11 ± 1.50b	8.96 ± 0.91a
	M–O	55.83 ± 4.74c	166.18 ± 7.84c	17.69 ± 0.70c		
	PC + M–O	59.70 ± 5.80c	175.82 ± 7.00c	19.21 ± 0.60d		
	PC–M	60.07 ± 9.82c	186.87 ± 13.5a	18.99 ± 1.60d		
**Moneymaker**	O–O	47.80 ± 3.94a	200.15 ± 13.0a	20.90 ± 1.30a	19.51 ± 0.80a	6.17 ± 0.68a
	PC–O	50.10 ± 3.45b	256.55 ± 19.4b	23.27 ± 1.10b	24.45 ± 1.10b	7.99 ± 0.68b
	M–O	43.37 ± 3.39c	162.38 ± 5.15c	15.62 ± 1.20c		
	PC + M–O	45.50 ± 3.93d	182.20 ± 7.73d	17.75 ± 0.30d		
	PC–M	46.07 ± 4.32d	189.46 ± 6.88d	17.87 ± 0.50d		
**NahG**	O–O	47.20 ± 3.20a	175.51 ± 10.5a	16.71 ± 1.10a	15.46 ± 1.20a	4.33 ± 0.29a
	PC–O	48.37 ± 3.21a	264.87 ± 7.52b	16.09 ± 0.90a	15.29 ± 0.40a	5.00 ± 0.67b
	M–O	44.43 ± 2.53b	160.41 ± 5.24a	15.43 ± 0.60b		
	PC + M–O	45.43 ± 3.66b	144.02 ± 5.88c	15.11 ± 0.90b		
	PC–M	45.30 ± 3.66b	140.80 ± 7.70c	13.64 ± 0.90c		
**Castlemart**	O–O	54.47 ± 5.41a	176.06 ± 7.22a	16.89 ± 0.600a	18.90 ± 0.70a	4.33 ± 0.25a
	PC–O	60.60 ± 4.90b	205.95 ± 8.32b	21.80 ± 1.20b	23.51 ± 1.00b	6.92 ± 0.56b
	M–O	49.87 ± 4.94c	155.47 ± 7.82c	14.13 ± 0.40c		
	PC + M–O	54.13 ± 5.41a	156.22 ± 9.11c	15.27 ± 0.60d		
	PC–M	56.17 ± 9.10a	152.66 ± 6.71c	15.28 ± 0.60d		
**Def1**	O–O	55.73 ± 5.21a	163.33 ± 11.90a	15.80 ± 0.80a	15.30 ± 0.80a	4.55 ± 0.46a
	PC–O	51.93 ± 5.05b	152.30 ± 11.80b	14.07 ± 0.60b	14.02 ± 0.50b	4.20 ± 0.32a
	M–O	48.90 ± 5.59b	139.46 ± 6.64c	13.94 ± 0.80b		
	PC + M–O	49.83 ± 6.77b	137.97 ± 7.69c	14.23 ± 0.60b		
	PC–M	41.57 ± 7.04b	138.71 ± 6.05c	13.62 ± 0.70c		

The split-root experiments consisted of five main treatments: O–O: the left and right root systems were not treated; PC–O: the left root system was pre-inoculated with PC-170 but the right was not treated; M–O: the left root system was challenged with *Meloidogyne incognita* but the right was not treated; PC + M–O: the left root system was pre-inoculated with PC-170 and then challenged with *M. incognita*, but the right was not treated; PC–M: the left root system was pre-inoculated with PC-170 and the right was challenged with *M. incognita*. At 45 dai with nematodes, plant height and root and shoot weight were measured. Each value is the mean ± SE ($n = 30$). Different letters indicate statistical differences between isolates ($p < 0.05$).
